# Human papillomavirus-16 is integrated in lung carcinomas: a study in Chile

**DOI:** 10.1038/sj.bjc.6603848

**Published:** 2007-06-19

**Authors:** F Aguayo, A Castillo, C Koriyama, M Higashi, T Itoh, M Capetillo, K Shuyama, A Corvalan, Y Eizuru, S Akiba

**Affiliations:** 1 Division of Oncogenic and Persistent Viruses, Center for Chronic Viral Diseases, Kagoshima University Graduate School of Medical and Dental Sciences, 8-35-1 Sakuragaoka, Kagoshima, 890-8544, Japan; 2 Department of Epidemiology and Preventive Medicine, Kagoshima University Graduate School of Medical and Dental Sciences, 8-35-1 Sakuragaoka, Kagoshima 890-8544, Japan; 3 Department of Human Pathology, Kagoshima University Graduate School of Medical and Dental Sciences, 8-35-1 Sakuragaoka, Kagoshima 890-8544, Japan; 4 Ijuin Hospital, 156 Tokushige, Ijuin 899-2502, Japan; 5 Departmento de Anatomia Patológica, Instituto de Anatomía Patológica, Hospital del Salvador, Av. Salvador 364, Providencia, Santiago, Chile; 6 Departamento de Patología, Pontificia Universidad Católica de Chile, 85 Lira street, Santiago 133-202, Chile

**Keywords:** human papillomavirus, viral load, integration, lung carcinoma, Chile

## Abstract

The human papillomavirus (HPV) was detected in 20 (29%) out of 69 lung carcinomas (LCs) in Chile, by PCR and Southern blot, and was more frequently detected in squamous cell carcinoma (SQC) than in adenocarcinomas (46 *vs* 9%, *P*=0.001). HPV-16, positive in 11 cases, was the most frequently detected HPV genotype determined by DNA sequencing. HPV-16 E2/E6 ratio, estimated from real-time PCR analysis, was much lower than the unity, suggesting that at least a partial HPV-16 genome was integrated in all but one HPV-16-positive SQCs. The remaining one case was suspected to have only episomal HPV-16. Although the viral load was low in most of the LCs, a case showed the HPV-16 copy number as high as 8479 per nanogram DNA, which was even a few times higher than the minimum viral load of seven cervical carcinomas (observed viral load: 3356–609 392 per nanogram DNA). The expression of the HPV-16/18 E6 protein was found in only two HPV-16-positive SQCs (13%) but not in the case with the highest viral load. Although the viral load was in general very low and HPV E6 expression is none or weak, further studies seem warranted to examine aetiological involvement of high-risk HPV in lung carcinogenesis.

The human papillomavirus (HPV) is an epitheliotropic double-stranded DNA virus, and its high-risk genotypes, including HPV-16 and -18, are associated with cancer of the uterine cervix and other genital cancers (Bosch *et al*, 2002; Zur Hausen, 2002). Human papillomavirus is also reported to be detected in lung carcinomas (LCs). A recent review (Syrjänen, 2002) of all the studies reported until 2001 showed that HPV was detected in 536 out of 2468 (22%) bronchial carcinomas.

The carcinogenic mechanism of HPV in genital cancers involves molecular alterations caused by its oncogenic products, such as E6 and E7, whose expressions are regulated by cis-acting elements in upstream regulatory region (Howley and Lowy, 2001). The E6 protein of HPV interacts with the p53 tumour suppressor protein and an E6-associated protein, a host cell ubiquitin ligase, and induces accelerated proteasomal degradation of p53 (Barbosa, 1996). Another study showed an inverse relationship between the presence of HPV DNA and abnormal p53 accumulation in LCs (Soini *et al*, 1996). However, the overexpression of p16^INK4a^ is known to be observed in cancers of the uterine cervix (Schorge *et al*, 2004), which are almost always associated with HPV infection (Bosch *et al*, 2002). More importantly, high-risk HPV was associated with a stronger expression of p16^INK4a^ than low-risk HPV (Sano *et al*, 1998). The binding of HPV E7 with p105Rb is considered to result in the release of E2F factors, which induces the high level of expression of p16^INK4a^ (Khleif *et al*, 1996).

In cervical cancer, the loss of the functional E2 gene is known to be one of the major genetic changes facilitating transformation and the transition into a malignant state (McBride *et al*, 1991; Hoppe-Seyler and Butz, 1994). In general, high-risk HPVs were frequently found to be integrated in cervical cancer and low-risk HPVs were frequently found to be episomal in benign lesions (Hudelist *et al*, 2004). In cervical cancer, integration of high-risk viral DNA into the host genome is an important but not essential event promoting carcinogenesis (Ho *et al*, 2006). When HPV integration occurs, it promotes disruption of the HPV E2 gene, leading to abnormal expression of E6 and E7 oncoproteins (Arias-Pulido *et al*, 2006).

A Chinese study examining oesophageal squamous cell carcinomas (SQCs) showed that only 9% out of 35 HPV-16-positive specimens exclusively harboured the episomal form, whereas the remaining 91% contained either only the integrated form (6%) or a mixture of episomal and integrated forms of viral molecules (86%). Among the 30 cancer specimens carrying mixed integrated and episomal forms, 28 had E2/E6 ratios of less than 1, indicating the presence of an integrated form of viral genes in these lesions (Si *et al*, 2005). Human papillomavirus can also be detected in cancers of the upper aerodigestive tract, including cancers of the oral cavity, larynx, and oesophagus tract. A study in Finland (Koskinen *et al*, 2003) reported HPV integration in cancers of the hypopharynx, larynx, tongue, and oral cavity. To our knowledge, no study has addressed the question as to whether HPV detected in LC exists in an episomal form or in an integrated form.

In the present study, we examined lung SQCs and adenocarcinomas (Acs) for HPV presence. Using real-time PCR (Peitsaro *et al*, 2002), we determined the HPV-16 load in SQCs and the integration status. To our knowledge, this is the first LC study to examine HPV viral load and integration in lung cancer specimens. In addition, we analysed the expression of the p16^INK4a^ and p53 proteins using immunohistochemistry.

## MATERIALS AND METHODS

### Study subjects

We examined 69 bronchoscopic biopsy specimens of paraffin-embedded tissue of SQCs (*N*=37) and ACs (*N*=32) of the lung, diagnosed in the Hospital Salvador, Santiago, Chile during the period between 1998 and 2003. According to the 1982 WHO classification of lung tumours, LC can be classified into four broad categories: SQCs, ACs, small cell carcinomas (SCCs), and large cell carcinomas (World Health Organization, 1982). In the present study, histological classification was made using the guideline of the Japan Lung Cancer Society (The Japan Lung Cancer Society, 2000), which follows the WHO classification. In addition, as positive control, we examined 20 carcinomas of the uterine cervix (18 SQCs and 2 adenosquamous cell carcinomas), diagnosed at Hospital Salvador in Santiago, Chile in 2000. Mean age and standard deviation of these cases were 52.1 and 17.0 years, respectively. The institutional review board of the Kagoshima University Graduate School of Medical and Dental Sciences of Japan approved the present study.

### Polymerase chain reaction, Southern blot and sequencing

Sections (10 *μ*m) of each formalin-fixed paraffin-embedded sample were prepared. We used 1–5 sections for each sample. The samples were added with 1 ml of xylene, and then with 1 ml of ethanol. After centrifugation, the pellet was resuspended in digestion buffer (50 mM Tris-Cl pH 8.0, 1 mM EDTA pH 8.0, 0.5% Tween 20) containing 200 *μ*g of proteinase K (Invitrogen Corp, Carlsbad, CA, USA) and incubated during 24 h at 56°C. Then the solution was heated at 100°C for 10 min and centrifuged at 10 000 r.p.m. for 10 min. Phenol–chloroform extraction was made in all the samples and the DNA was precipitated with double volumes of ethanol. The DNA was quantified using ND-1000 Spectrophotometer (Nano Drop Technologies, Wilmington, USA). Since the quantity of tissue embedded in the paraffin blocks was variable between samples, *β*-globin amplification was made for all the samples to check the presence of PCR amplification inhibitors and of amplifiable DNAs. *β*-Globin amplification with a set of PCO3/PCO4 primers (Takara, Otsu, Japan) was conducted under the following PCR conditions: initial denaturation at 95°C for 4 min, 40 cycles with the cycling profile of 95°C for 1 min, 52°C for 1 min and 72°C for 2 min and final extension for 5 min at 72°C. All the samples were positive for a fragment of the *β*-globin gene.

Human papillomavirus amplification with GP5+/GP6+ primers (De Roda Husman *et al*, 1995) was made in a reaction mixture that contained 2.5 *μ*l of template DNA, 200 *μ*M dNTP, 0.5 *μ*M of each primer and 1.0 U Taq DNA polymerase (Takara) in a total volume of 25 *μ*l of reaction buffer (50 mM KCl, 20 mM Tris-Cl, pH 8.3). The conditions of amplification were as follows: initial denaturation at 95°C for 4 min; subsequent 45 cycles at 95°C for 1 min, 40°C for 2 min and 72°C for 1.5 min and final extension at 72°C for 5 min. HPV-6 and -18 full genomes (kindly given by Dr H zur Hausen, Germany) were used as the external positive control. The amplified products were confirmed through electrophoresis with 3.0% agarose gel. Previous to the sequencing, Southern blot was conducted to confirm the identity of amplified fragments and to increase the sensitivity of detection of amplified fragments. As for HPV probes, we used cloned PCR products of the HPV-positive DNAs, which were kindly given by Dr H zur Hausen, Germany, after purification of the PCR products from agarose gel by QIAEX II Extraction Kits (Qiagen GmbH and Qiagen Inc., Hilden, Germany). The ECL direct labelling and detection kit (Amersham, Buckinghamshire, UK) was used according to the manufacturer's instructions. An ECL-labelled HPV18/GP5+/GP6+ fragment was used to detect high-risk HPVs and an ECL-labelled HPV6/GP5+/GP6+ fragment was used to detect low-risk HPVs. Because the generic HPV18 and -6 probes were used, low stringency conditions were used in the hybridizations and washes. The DNA was transferred onto a Hybond N+ nylon transfer membrane (Amersham) by capillary blotting using 0.4 N NaOH. The hybridization was made at 42°C overnight and then the membranes were washed at 42°C with solution containing 6 M urea, 0.4% SDS and 0.5 X SSC buffer. The samples that showed visible bands in the Southern blot procedure but not in the agarose gel electrophoresis were amplified again using 1/100 dilution of the first PCR product using GP5+/GP6+ protocol in 30 cycles. Negative controls were used to confirm no carryover of amplicons. After double PCR using GP5+/GP6+ primers, the obtained fragments were purified and sequenced to examine the genotype of additional HPV-positive samples by Southern blot analysis. In all the steps, technical considerations were taken, to avoid carryingover and contamination with previous amplicons in the tubes.

The HPV genotyping was made using DNA sequencing. Amplified PCR products that appeared as a visible band after ethidium bromide staining were purified using the QIAGEN PCR purification kit and were directly sequenced by fluorescent dye-labelled dideoxynucleotides and cycle sequencing methods using the Big DyeTerminator Cycle Sequencing Kit (PE Applied Biosystems, Foster City, USA). Sequence analysis was performed on the ABI PRISM 310 Genetic Analyser (PE Applied Biosystems). The nucleotide sequences were aligned and compared with those of known HPV types available through the GenBank database (National Institute of health, USA) by using BLAST 2.2 (http://www.ncbi.nih.gov/BLAST/) and ClustalW (http://clustalw.genome.jp/) software servers.

### Immunohistochemistry for p16, p53, and HPV-16/18 E6

The paraffin-embedded samples were cut in 2–3 *μ*m-thick slices, deposited in coated glass slides, and dewaxed using xylene. After rinsing with ethanol, the slides were incubated 30 min in 0.3% H_2_O_2_/methanol and 5 min in microwave at 95°C in 0.01 M sodium phosphate/citrate buffer (pH 8.0). In order to block the nonspecific binding of the antibody, the slides were incubated for 30 min with 5% bovine seroalbumin (BSA) in PBS at room temperature. A 1/200 dilution in 5% BSA–PBS of monoclonal anti-p16^INK4a^ antibody was used (BD PharMingen, San Jose, USA). The slides were incubated overnight at 4°C, washed with PBS, and then incubated with biotinylated horse anti-mouse Ig-G for 30 min followed by washing with PBS and incubation with 1 : 50 dilution of the avidin–biotin–peroxidase complex (Vectastain Elite ABC kit, Vector Laboratories, Burlingame, USA) for 30 min at room temperature. The reaction was visualized by adding diaminobenzidine (Dako Corporation, Carpinteria, USA) for 10 min. The sections were counterstained with hematoxylin and visualized. Negative reaction was considered when 0–9% of the cells were stained. Positive reaction was considered when 10–100% of the cells were stained, according to criteria reported previously (Cheng *et al*, 2003). For p53 and HPV-16/18 E6 immunostaining, the procedure was the same as it was for p16^INK4a^, but primary antibodies of p53 (1 : 50 dilutions) and HPV-16/18 E6 (1 : 50 dilutions) were used (DO-7, Dako Japan Co. Ltd., Kyoto, Japan, and C1P5, Santa Cruz Biotechnology Inc., Santa Cruz, USA, respectively). The interpretation of the positive signal was the same as that used for p16^INK4a^ immunostaining.

### Real-time PCR

To estimate viral load and to determine the integration status of HPV-16, real-time PCR was performed with the ABI Prism 7000 Sequence Detection System and TaqMan Universal PCR Master Mix (Applied Biosystems, Roche Molecular Systems, Foster City, USA). All but one the HPV-16-positive specimens confirmed by DNA sequencing were subjected to the analysis. The analysis of the remaining one sample was not possible because its paraffin-embedded tissue specimens were exhausted. The amplification conditions were 2 min at 50°C, 10 min at 95°C, and a two-step cycle of 95°C for 15 s and 60°C for 60 s for a total of 45 cycles (Peitsaro *et al*, 2002). The primers used were: E6F: gagaaactgcaatgtttcaggacc and E6R: tgtatagttgtttgcagctctgtgc, E2F: aacgaagtatcctctcctgaaattattag and E2R: ccaaggcgacggctttg that amplify a fragment of 81 and 76 bp of E6 and E2 ORFs, respectively (Peitsaro *et al*, 2002). We used the E6F/R primer set for viral load and both E2F/R and E6F/R primer sets for integration detection. The probes used were caccccgccgcgacccata for E2 and caggagcgacccagaaagttaccacagtt for E6 (Peitsaro *et al*, 2002). Both E2 and E6 probes were labelled with FAM at the 5′ end and with TAMRA at the 3′ end (Applied Biosystems Japan Ltd., Tokyo, Japan). The PCR amplification was performed in a 25 *μ*l volume containing TaqMan 2 × PCR Master Mix with 0.5 *μ*M of E2- or E6-specific primers, 100 nM of dual-labelled E2 or E6 fluorogenic hybridization probe, and 1 *μ*l of DNA template. For specificity and validity testing, DNAs from SiHa celll line (Mincheva *et al*, 1987), which is known to contain one HPV-16 copy or two per cell, were also examined as templates. Two standard curves for the E2 and E6 fragments were made by amplification of dilutions between 862 million to 86 copies of HPV-16 cloned in pUC19 plasmid (kindly given by Dr Massimo Tommasino, IARC, France). There was a linear relationship between the threshold cycle values plotted against the log of the copy number over the entire range of dilutions. All the experiments were made in duplicate.

PCR amplifications were observed correctly when the target strains were used as templates. The primers and probes did not increase the fluorescence with the mismatched strains (data not shown).

In order to determine the physical status of HPV-16, the ratio of E2 to E6 copy numbers was calculated (Peitsaro *et al*, 2002). Since E2 is degraded when HPV is integrated (Jeon and Lambert, 1995) into cellular genome, E2/E6 ratio nearly equal to 1 indicates the presence of the episomal form only. Values less than 1 indicate the presence of both integrated and episomal forms, while a ratio of 0 indicates that the fragment was not detected after 45 cycles of amplification and that HPV exists in the integrated form only.

We also conducted real-time PCR for *β*-globin (110 bp) to determine the genome equivalent present in each sample and to normalize samples for genomic DNA content. A 10-fold dilution series of a human DNA control (Dynal UK Ltd, Wirral, UK) was used to generate the standard curve for *β*-globin. The amount of *β*-globin DNA present in each sample was divided by the weight of one genome equivalent (i.e. 6.6 pg cell^−1^) and a factor of 2 (since there are two copies of *β*-globin DNA/genome equivalent) to obtain the number of genome equivalents in each sample. Viral load in each specimen was expressed as the number of HPV copies/genome equivalent or cell.

### Statistical analysis

Fisher's exact test was used to examine the statistical significance of the results. *P*-values presented are two sided.

## RESULTS

We examined 37 SQCs and 32 ACs of the lung diagnosed in Santiago, Chile. Their clinicopathological features are summarized in Table 1. Squamous cell carcinomas were more common among men than among women (*P*=0.007), and also more common among smokers (*P*=0.013). The frequency of p16^INK4a^ or p53 expression did not show any significant difference between SQCs and ACs. The expression of p16^INK4a^ was positive in 24 out of 30 in well- or moderately differentiated SQCs but in only 1 out of 6 poorly differentiated SQCs (*P*=0.008). Such a difference was not observed in ACs. The expression of p53 was related to tumour differentiation in neither SQCs nor ACs.

The results of HPV detection and genotypes determined by DNA sequencing in LC are summarized in Table 2. Human papillomavirus was detected in 17 (46%) and 3 (9%) of SQCs and ACs, respectively. The difference of HPV prevalence between SQCs and ACs was statistically significant (*P*=0.001). This difference was mainly accounted for by high-risk HPVs, which were detected in 16 SQCs and in 1 AC. Human papillomavirus presence was not related to gender, age, or smoking habits (data not shown). Even when age was divided into finer categories, we could not observe any evident relationship between age and HPV presence. The expression of p16^INK4a^ or p53 showed no significant difference between HPV-positive and -negative LCs, either.

As shown in Table 2, the most frequent HPV genotype was HPV-16, which was detected in 10 SQCs and 1 AC. In addition, we analysed 18 SQCs and 2 adenosquamous cell carcinomas of the uterine cervixes from Chile, using the same methodology as shown in Table 2. Among the 18 SQCs, there were 11 well- or moderately differentiated SQCs and 7 poorly differentiated SQCs. Human papillomavirus was detected in 19 out of 20 (95%) cervical cancer samples. We could not detect HPV genome in one case with adenosquamous cell carcinoma. All the HPV-positive cases harboured high-risk HPVs, including HPV-16. In two HPV-16- and one HPV-59-positive cases, we also detected the low-risk type HPV-6/11.

We conducted real-time PCR analysis to estimate viral load and to determine the integration status of HPV-16 (Table 3). All but one the HPV-16-positive specimens were subjected to the analysis. The analysis of the remaining one sample was not possible because its paraffin-embedded tissue specimens were exhausted. The validity of the assays was confirmed by accurate quantification of viral copy numbers (one copy per cell) from SiHa cell line, which is known to contain one HPV-16 copy or two per cell. The median viral loads of lung SQCs and cervical cancer specimens were 8.2 and 64 254 copies per nanogram of DNA, respectively. SiHa cell line had 144 E6 copies per nanogram of genomic DNA. Most of the lung SQCs examined had E6 copies less than this figure. However, there was one case (LC-5) having E6 copies per cell as large as 8479, a much larger figure than that of SiHa cell line. The case LC-5 was a 75-year-old male smoker, and the tumour was well-differentiated type SQCs with p16^INK4a^ and p53 expression in 10 and 70% of tumours, respectively. We also examined 7 out of 12 HPV-16-positive cervical carcinomas, and three of them had E6 copy numbers lower than that of the case LC-5. Viral load was not related to any clinicopathological features examined (data not shown).

E2/E6 ratios are presented in Table 3. All the cervical cancer cases had an E2/E6 ratio less than 0.02, indicating to harbour integrated HPV-16. In LC cases, there were two cases (LC-1 and -5) where E2 could not be detected and four cases where E2 copy number was lower than detectable level, less than 1 × 10^−6^ ng DNA *μ*l^−1^. Those six LC cases were considered to have only integrated form of HPV. On the other hand, there were two cases where E2/E6 ratio was 0.01–0.02 and considered to have both integrated and episomal forms of HPVs (integration status: mixed). The remaining one case had the E2/E6 ratio of about one, suggesting the episomal form.

**Figure 1:**
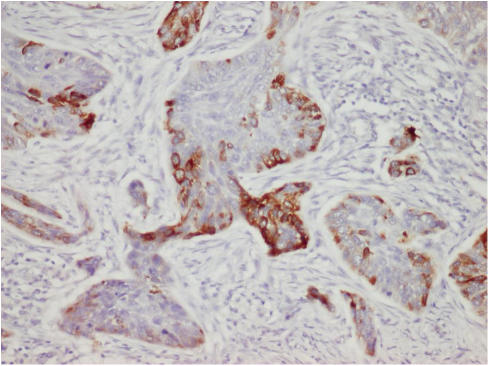
HPV-16/18 E6 immunostaining. The E6 expression was observed in cytoplasm of tumour cells (× 200).

We also examined the E6 expression among 14 HPV-16/18-positive cases by immunohistochemistry. There were only two SQC cases (13%) showing the E6 expression, and both of them were HPV-16 positive (Table 3). Case LC-5, having the highest viral load, did not show E6 expression. The E6 expression was observed in the cytoplasm of partial tumour cells (Figure 1). Neither the copy number of E6 nor HPV-integration status was related to the E6 expression (Table 3). These E6-positive SQCs were negative for p53 expression.

## DISCUSSION

In the present study, we detected HPV in 46% of SQCs but in only 9% of ACs of the lung. The difference between SQCs and ACs was highly significant (*P*=0.001). Among HPV genotypes detected, 85% were high-risk HPV. The most frequently detected genotype in the present study was HPV-16, which accounted for 55% of HPV-positive cases, as was also the case in our previous study conducted in other Latin-American countries, including Mexico, Colombia, and Chile (Castillo *et al*, 2006). Our findings are also in accordance with those shown in a review published by Syrjänen (2002), where HPV was positive in 25% of SQCs, 19% of SCCs, and 8% of ACs.

Physical status of HPV was determined by HPV E2/E6 ratio in the present study, and most of the HPV-16-positive SQCs were considered to have HPV-16 in the integrated form, and about half of the integrated HPV-16 was accompanied by episomal HPV-16. Note, however, that this approach first reported by Peitsaro *et al* (2002) does not directly show the presence of integrated HPV in the cellular genome by, for example, its sequencing. However, our observation that E2/E6 ratio did not become larger than the unity indirectly supports the validity of this approach.

Although the viral loads of most LCs were low, one case showed the viral load of HPV-16 as high as 8479 ng^−1^ DNA, which was even higher than minimum viral load of the seven cervical carcinomas (viral load: 3356–609 392 per nanogram DNA) examined in the present study. Since SiHa cell line, having 1–2 HPV copies per cell, was found to have 144 copies per nanogram DNA in our analysis, this LC case is suspected to have HPV copies much more than 1–2 per cell.

If a clonal expansion occurs after HPV infection in lung epithelial cells, HPV is expected to be found in all the carcinoma cells. However, our results suggest that this scenario is unlikely because only a very small proportion of the malignant cells are integrated. Regarding the role of HPV in LCs, Kinjo *et al* (2003) has argued that HPV is not aetiologically involved in LC development but that HPV induces squamous metaplasia in ACs. Another possible explanation for such a low viral load may suggest a ‘hit-and-run’ mechanism, where the virus DNA may be lost after transformation, as shown in studies on a bovine model, and as suggested in HPV-18 oncogenesis and in non-melanoma skin cancer developments mediated by HPV (Iwasaka *et al*, 1992; Aubin *et al*, 2003).

The integration of HPV in the LC genome does not necessarily mean that HPV is involved in the development of lung cancer. In order to examine the expression of proteins that are considered to be associated with altered signal pathways in cervical cancer, we compared the expression of suppressor genes in HPV-positive and -negative LCs. In cervical carcinomas, p16^INK4a^ is known to be upregulated by HPV (Ishikawa *et al*, 2006). In the present study, however, p16^INK4a^ was expressed in more than 80% of lung SQC cases regardless of HPV presence and we could not find any evidence suggesting p16^INK4a^ upregulation by HPV in LCs. A study reported by Geradts *et al* (1999) has shown that about 50% of non-small cell LCs are with positive p16^INK4a^ expression, which was defined as a diffuse or mosaic pattern throughout a tumour. They also pointed out variable p16^INK4a^-positive frequencies in different reports, possibly due to genetic backgrounds, environmental factors, biological heterogeneity, and/or technical differences. Therefore, it seems difficult to make comparison between different studies. In our previous study of gastric cancer in Japan, 60% of them were p16^INK4a^ positive (Koriyama *et al*, 2004). Taken together, LCs may have higher p16^INK4a^ expression than other cancers. In the case of cervical cancer, the p105Rb is sequestered and degraded by HPV E7, causing the release of E2F protein, which in turn leads to p16^INK4a^ upregulation. In LC, other mechanisms may be at work.

The p53 gene is reported to be frequently mutated in lung SQCs (Sanchez-Cespedes, 2003), and missense mutations frequently cause the abnormal accumulation of p53 (Toyooka *et al*, 2003). A large-scale study showed that about 52% of lung SQCs was p53 positive (Tammemagi *et al*, 1999). In the present study, p53 was positive in less than 50% of carcinomas regardless of HPV presence. When the proportion of p53-positive cells in each carcinoma was examined (data not shown), we found no difference between HPV-positive and -negative SQCs. The HPV-16/18 E6 protein has been reported to interact with p53 and target it for degradation through an ubiquitin-dependent pathway (Scheffner *et al*, 1990). Although the number of E6-positive cases was small in the present study, all of them were p53 negative.

It is suspected that HPV was more strongly associated with well-differentiated SQCs, featured by conspicuous pearl formation, than less-differentiated SQCs (Miyagi *et al*, 2000). A Greek study also showed such an association (Papadopoulou *et al*, 1998). We also observed pearl formation in 4 out of 8 well-differentiated SQCs (50%). However, two of them were HPV-16 positive and the rest were HPV negative (data not shown).

A large study on numerous fresh frozen LCs in West-European countries found a low prevalence of HPV16 and a very low viral load without any HPV16 E6 mRNA expression (Coissard *et al*, 2005). On the other hand, studies in Latin-American and Asian LCs reported relatively high prevalence of HPV, including HPV16 (Miyagi *et al*, 2000, Castillo *et al*, 2006) even though they used formalin-fixed paraffin-embedded carcinoma specimens.

The DNA specimens extracted from paraffin-embedded blocks are known to be frequently degraded into 200 bp long or even shorter fragments (Ben-Ezra *et al*, 1991; Karlsen *et al*, 1994). For this reason, we used GP5+/GP6+ primers for PCR to detect a short fragment from the L1 region of HPV (155 bp) (De Roda Husman *et al*, 1995). In addition, all of the samples we examined were *β*-globin positive, which is 110 bp. In the present study, amplified products by PCR were analysed by Southern blot hybridization using the ECL-labelled probes obtained from cloned GP5+/GP6+ amplified products of HPV-positive control DNAs. When compared to PCR-agarose gel electrophoresis and *in situ* hybridization (data not shown), our method is considered to be more sensitive and enables us to detect HPV even when carcinoma cells have a small number of HPV copies. However, it is not possible for us to tell whether HPV is present in tumour or normal cells because most tissue specimens contained adjacent normal cells. Further studies using the microdissection technique are necessary to address this question.

We detected HPV-18 in three SQC cases but not in ACs. In the uterine cervix, HPV-18 has been shown to have a higher risk of developing Acs, while HPV-16 is more strongly associated with SQCs (Castellsague *et al*, 2006). In the case of the lung, the association of HPV-18 with ACs was shown by a study of non-smoking Taiwanese women (Cheng *et al*, 2001). A Latin-American study also detected HPV-18 only in lung ACs (Castillo *et al*, 2006). A recent meta-analysis by Chen *et al* (2004) showed that 9–42% of pulmonary ACs among Asians were HPV positive. However, HPV-18 was not detected exclusively in ACs. For example, a North American study reported by Bohlmeyer *et al* (1998) detected HPV-18 in 2 out of 34 lung SQCs. Interestingly, their study did not find any other HPV genotypes. Another study, conducted in China, detected both HPV-16 and -18 in ACs of the lung at similar frequencies (Fei *et al*, 2006). At this moment, it is difficult to postulate that HPV-18 is more strongly related to ACs than SQCs of the lung.

The transmission route of the HPV detected in LCs is as yet unclear. Studies on HPV infection and cancers of the oral cavity, oesophagus and lung suggested the possibility of sexual transmission (Smith *et al*, 2004). Recent studies in Taiwan detected identical sequences of L1 and E6 of HPV16/18 in LCs and blood cells. In addition, female lung cancer patients showed a correlation between HPV16/18 detection frequencies in LCs and cervical smears (Chiou *et al*, 2003). Those findings suggest that HPV detected in LC may originate in the uterine cervix and spread to lung tissue via the bloodstream. However, a recent study in Latin America, which compared the second cancer risk of 335 women with invasive cervical cancer and their first degree relatives, did not find any increase in LC risk among cervical cancer patients (Weber *et al*, 2005).

In summary, high-risk HPV, including HPV-16, was detected in Chilean SQC cases at a high frequency, and the real-time PCR analysis suggested the integration of HPV-16 into the cellular genome of SQC specimens. Although the viral load was in general very low and HPV E6 expression is none or weak, further studies seem warranted to examine aetiological involvement of high-risk HPV in lung carcinogenesis.

## Figures and Tables

**Table 1:** Clinicopathological features of lung SQC and AC

	**Number of subjects (%)**		
	**SQC**	**AC**	*P*-value*
Total	37 (100)	32 (100)	
*Gender*			0.007
Male	32 (86)	18 (56)	
Female	5 (14)	14 (44)	
			
*Age (years)*			0.415
−64	12 (32)	15 (47)	
65−	20 (54)	15 (47)	
75+	5 (14)	2 (6)	
			
*Smoking*			0.013
Non smoker	2 (5)	10 (31)	
Smoker	29 (78)	20 (63)	
Unknown	6 (16)	2 (6)	
			
*Tumour differentiation*			0.441
Well	8 (22)	7 (22)	
Moderate	23 (62)	16 (50)	
Poor	6 (16)	9 (28)	
			
*P16* a			0.799
Negative (−9%)	11 (31)	11 (34)	
Positive (10%−)	25 (69)	21 (66)	
			
*P53* a			0.809
Negative (−9%)	20 (56)	19 (59)	
Positive (10%−)	16 (44)	13 (41)	

SQC= squamous cell carcinoma; AC=adenocarcinoma.

^*^P-values for difference between SQCs and ACs were obtained from Fisher's exact test.

a Expression of p16 and p53 was not examined in one SQC because of the shortage of tissue sample.

**Table 2:** HPV detection in lung and cervical cancers

	**Number of subjects (%)**			
	**Cervical**	**Lung cancer**		
	**cancer**	**SQC**	**AC**	***P*-value^a^**
Total	20 (100)	37 (100)	32 (100)	
*HPV positive cases*				
All HPV genotype	19 (95)	17 (46)	3 (9)	0.001
High-risk HPV^*^				
HPV-16	12 (60)	10 (27)	1 (3)	0.008
HPV-18	2 (10)	3 (8)	—	0.243
HPV-31	—	1 (3)	—	1.000
HPV-33	1 (5)	—	—	
HPV-45	3 (15)	2 (5)	—	0.495
HPV-59	1 (5)	—	—	
				
Low-risk HPV^*^				
HPV-6	—	1 (3)	2 (6)	0.593
HPV-6/11	3 (15)^b^		—	—

HPV=human papillomavirus; SQC=squamous cell carcinoma; AC=adenocarcinoma.

^*^Classification of high- and low-risk HPV genotypes was based on the report by Munoz *et al*.

a *P*-values for difference between SQCs and ACs of the lung were obtained from Fisher's exact test.

b Co-infection with high-risk HPV was observed in two SQCs (moderately or poorly differentiated) and one adenosquamous cell carcinoma.

**Table 3:** HPV-16 viral load, physical status, and immunostaining for E6 in lung SQCs and cervical carcinomas

	**Viral load**		**Integration**		**Immunohistochemistry**		
	**E6/ng of DNA**	**HPV-16 copies/cell**	**E2/E6**	**Status**	**HPV-16/18 E6^a^**	**p16^a^**	**p53^a^**
*Lung cancer*							
LC-1	4 × 10^−2^	1 × 10^−7^	No E2^b^	Integrated	−	+	−
LC-2	51	0.1	0.02	Mixed	−	+	−
LC-3	74	0.3	0.01	Mixed	−	+	−
LC-4	4	0.4	<1 × 10^−6^	Integrated	+	−	−
LC-5	8479	217	No E2^b^	Integrated	−	+	+
LC-6	207	1	<1 × 10^−6^	Integrated	−	+	−
LC-7	1	0.01	<1 × 10^−6^	Integrated	−	+	−
LC-8	4	1	<1 × 10^−6^	Integrated	−	+	+
LC-9	8.2	1 × 10^−4^	1.06	Episomal	+	+	−
							
*Cervical cancer*							
CX-3	79 827	29 174	0.02	Mixed			
CX-5	64 254	372	0.02	Mixed			
CX-6	609 392	425	0.02	Mixed			
CX-8	3495	1	0.02	Mixed			
CX-9	3356	2650	<1 × 10^−6^	Integrated			
CX-10	105 292	754	3 × 10^−3^	Mixed			
CX-11	5527	30	0.02	Mixed			
							
SiHa cell line	144	1	<1 × 10^−6^	Integrated			

a HPV-16/18 E6, p16, and p53 expressions were not examined in cervical carcinomas.

b HPV-16 E2 was not detected by TaqMan real-time PCR.
